# Exploiting tumor aneuploidy as a biomarker and therapeutic target in patients treated with immune checkpoint blockade

**DOI:** 10.1038/s41698-023-00492-8

**Published:** 2024-01-02

**Authors:** Liam F. Spurr, Sean P. Pitroda

**Affiliations:** 1 Pritzker School of Medicine, Biological Sciences Division, The University of Chicago, Chicago, IL USA; 2 Department of Radiation and Cellular Oncology, The University of Chicago, Chicago, IL USA; 3 Ludwig Center for Metastasis Research, The University of Chicago, Chicago, IL USA

**Keywords:** Cancer genomics, Tumour biomarkers

Since the advent of immune checkpoint blockade (ICB), there has been substantial interest in identifying biomarkers of response to facilitate treatment personalization for patients with various cancers. Tumor mutational burden (TMB) has received considerable attention as a validated biomarker of ICB response, which resulted in the 2020 FDA approval for the use of high TMB (≥10 mutations per megabase of DNA) as a tissue-agnostic biomarker for patients treated with pembrolizumab.

However, the overall response rate in TMB-high tumors in the study which catalyzed FDA approval was only 29%^1^. Thus, much debate remains regarding how to optimally utilize TMB as a biomarker of ICB response and whether other tumor or host features provide additional predictive value in this setting. Multiple complementary biomarkers have been proposed, including neoantigen load, CD8+ T cell expression signatures, PD-L1 expression, mismatch repair deficiency, and HLA genotype^2^.

Aneuploidy, an unbalanced chromosome arm copy-number, is a nearly ubiquitous feature of human cancer. In recent years, there have been significant efforts to understand its role in the generation and propagation of malignant cells. Multiple studies have proposed that aneuploidy may exert an oncogenic effect partially through suppression of anti-tumor immunity. Davoli et al.^3^ showed that aneuploidies, as opposed to focal copy-number alterations, were specifically associated with reduced expression of an immune signature representative of CD8+ and natural killer T cell function, which the authors hypothesized could be due to protein imbalance. Aneuploidies, by definition, affect the copy-number, and therefore, potentially the expression of a substantial number of genes. It is possible that some aneuploidies impact a cancer cell’s ability to produce proteins involved in signaling required for effective tumor immune infiltration and response to immunotherapy. These observations have since been buttressed by findings from Alessi et al. that higher degrees of aneuploidy are associated with reduced cytotoxic immune cell infiltration^4^.

Recent works from our group and others have found additional support for the notion that tumor aneuploidy is a predictor of poor survival after treatment with immunotherapy across multiple cancer types^4–6^. Specifically, aneuploidy provides prognostic value which is synergistic with TMB: patients whose tumors had a low TMB exhibited the most deleterious prognosis with a high level of aneuploidy.

Still, an important question is how to exploit tumor aneuploidy for therapeutic benefit. Girish et al. recently demonstrated that tumor cells can become “addicted” to specific aneuploidies, such as trisomy of chromosome 1q. In this context, increased copy-number of *MDM4* leads to decreased p53 activation, but simultaneously increases cellular dependence on triploid *UCK2*, a pyrimidine ribonucleoside kinase. The authors showed that this reliance on *UCK2* can in turn be exploited with cytotoxic nucleotide analogues, such as *RX-3117* and *3-deazauridine*^7^.

**Fig. 1:**
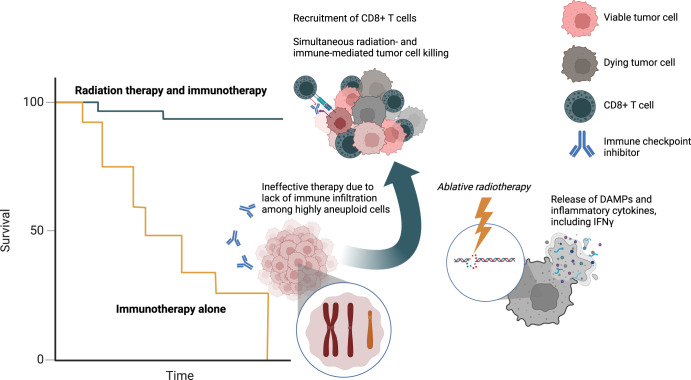
Proposed mechanism of immunotherapy response augmentation. High tumor aneuploidy is associated with poor survival following immunotherapy alone. However, the available evidence indicates that simultaneous administration of radiation therapy results in concurrent tumor cell killing and influx of immune cells. These synergistic effects lead to more effective tumor elimination and augmentation of systemic disease response and survival.

In addition to the targeting of tumor-specific aneuploidies, a complementary strategy is to target tumor cells with a high number of aneuploidies. We recently demonstrated that simultaneous administration of radiation therapy (RT) augments the response to immunotherapy in patients with highly aneuploid metastatic non-small cell lung cancer, but not those with tumors harboring a low degree of aneuploidy^8^. Our findings have additionally been shown to extend to patients who received chemoradiation therapy followed by immunotherapy for locally advanced NSCLC^9^. We posited that this observation may be due to synergistic activation of the anti-tumor immune response by combination RT and immunotherapy. As described above, highly aneuploid tumors often exhibit poor immune cell infiltration. RT has been demonstrated to enhance innate and adaptive immunity in preclinical studies through multiple mechanisms including (1) upregulation of the cytokine and chemokine production, (2) release of tumor DNA activating cGAS/STING signaling, induction of interferon signaling, and subsequent recruitment and activation of dendritic and CD8+ T cells, and (3) release of damage-associated molecular patterns which activate antigen presenting cells^10^. Thus, the use of local ablative RT may specifically attenuate the poor immune infiltration commonly present in highly aneuploid tumors, thereby enhancing local immunity and leading to improved responses to immune checkpoint blockade (Fig. 1). It is plausible that other cytotoxic agents, such as DNA-damaging chemotherapies, may induce a similar immune stimulatory effect. It is also reasonable to suggest that a subset of tumors with a low degree of aneuploidy who harbor low intratumoral immune cell content may benefit from combination RT and ICB. Adjunctive methods to characterize tumor-specific mechanisms of resistance could identify if such a subpopulation exists and could help to further select patients with highly aneuploid tumors most likely to derive a benefit from combination therapy.

In summary, we propose that while highly aneuploid tumors exhibit a worse baseline prognosis, they also present unique opportunities to advance the standard of care, both through development of new therapies and more effective use of existing ones. Understanding the role of aneuploidy in treatment resistance could create inspiration for development of agents targeting tumor-essential arm-level copy number aberrations. Moreover, we could leverage the secondary effects of cytotoxic therapies (including chemotherapy and radiation therapy) in patient populations where they are not routinely administered with immunotherapy. Importantly, in a similar manner as TMB, our work and that from other groups have shown that it is possible to quantify aneuploidy from clinical targeted panel sequencing assays, even from platforms which do not utilize matched normal tissue^4,5,8,9^ using tools such as ASCETS (Arm-level Somatic Copy-number Events in Targeted Sequencing)^11^. Thus, this biomarker can be readily applied from existing sequencing architecture which is routinely used to tailor systemic therapies. It is probable that other computational methods for identifying cancer aneuploidies would have utility in this setting as well. In an era with genomic sequencing and precision oncology becoming more commonplace, we have an important opportunity to leverage the most common alteration in human malignancies for the benefit of patients living with cancer.
